# Monocarboxylate Transporter‐1 Is Dispensable for Hepatocellular Carcinoma Development

**DOI:** 10.1002/mc.70021

**Published:** 2025-07-31

**Authors:** Shaimaa A. Gad, Bryan Bridgeman, Kyle Boedeker, Xianzhong Ding, Wei Qiu

**Affiliations:** ^1^ Departments of Surgery, Pathology Loyola University Chicago Stritch School of Medicine Maywood Illinois USA; ^2^ Departments of Cancer Biology, Pathology Loyola University Chicago Stritch School of Medicine Maywood Illinois USA; ^3^ Department of Pharmacology, Medical Research and Clinical Studies Institute National Research Center Egypt; ^4^ Department of Pathology Loyola University Chicago Stritch School of Medicine Maywood Illinois USA

**Keywords:** dispensable, hepatocellular carcinoma, metabolism, monocarboxylate transporter 1

## Abstract

Hepatocellular carcinoma (HCC) is the most prevalent type of liver cancer and the deadliest liver disease. It is imperative to understand the underlying molecular mechanisms involved in the development of HCC. Monocarboxylate transporter‐1 (MCT1) is a proton‐coupled protein that facilitates the bidirectional transport of monocarboxylates, such as lactate and pyruvate, across the plasma membrane to maintain the cellular metabolism and energy supply. MCT1 was found to be upregulated in human HCC specimens, and its inhibition reduced xenograft tumor growth. However, the role of MCT1 in HCC remains to be further investigated using immune‐competent in vivo models. To better understand the role of MCT1 in HCC, we established liver‐specific MCT1 knockout mice. We found that deletion of MCT1 in liver cells did not affect morphology, proliferation, or apoptosis. DEN/CCl4 model, where a single injection of DEN is followed by repeated injections of CCl4, was used to induce HCC in mice. Intriguingly, we found that liver‐specific knockout of MCT1 was not sufficient to reduce the size or count of DEN/CCl4‐induced liver tumors. In addition, we used immunohistochemical staining to evaluate the expression of Ki67, collagen A1, and myeloperoxidase, and we found that MCT1 knockout was not able to hinder the proliferation, fibrosis, and inflammation in the DEN/CCl4‐induced HCC tumors. In conclusion, MCT1 is dispensable for HCC development, and its deletion was insufficient to alleviate the phenotypic repercussions of HCC tumors in the DEN/CCl4‐induced HCC model.

**Abbreviations**
AFP: alpha fetoprotein
COLA1: collagen A1
DEN/CCl_4_
: diethylnitrosamine/Carbon tetrachloride
HCC: hepatocellular carcinoma
H&E: hematoxylin, and eosin
IHC: immunohistochemistry
MCT1: monocarboxylate transporter 1
MCT1 f/f: MCT1^flox/flox^
MPO: myeloperoxidase
α‐SMA: alpha smooth muscle actin

## 1 Introduction

Hepatocellular carcinoma (HCC) constitutes more than 85% of all primary liver cancers, making it a significant global health concern [1]. It ranks sixth in terms of common cancers and fourth in terms of cancer‐related fatalities worldwide. In the United States, there were an estimated 42,220 new cases and 30,200 deaths from liver cancer in 2018 [2]. Unfortunately, the prognosis for HCC patients is grim, with a 5‐year survival rate of less than 18% [3]. The only curative treatments for HCC are surgical resection or liver transplantation, which require early detection or the availability of a suitable donor, respectively—neither of which is common. Several first‐line and second‐line therapeutic agents are FDA‐approved for advanced HCC, such as sorafenib [4], lenvatinib [5], regorafenib [6], cabozantinib [7], nivolumab [8], and atezolizumab combined with bevacizumab [9]. However, these typically increase overall survival by only a matter of months. There is an urgent imperative need to unravel the molecular mechanisms underpinning HCC development to improve survival rates for HCC patients.

Monocarboxylate transporter 1 (MCT1), encoded by the SLC16A1 gene, is a proton‐linked transporter responsible for the bidirectional transmembrane movement of monocarboxylates such as lactate, pyruvate, and acetate [10, 11]. By regulating the flux of these metabolic intermediates, MCT1 plays a critical role in maintaining cellular energy homeostasis, PH balance, and metabolic adaptation under physiological and pathological conditions [11, 12]. In cancer, MCT1 is increasingly recognized as a key metabolic regulator that supports the high energetic and biosynthetic demands of rapidly proliferating tumor cells [12, 13]. Elevated expression of MCT1 enables efficient lactate export from glycolytic cancer cells and acetate import in nutrient‐deprived environments, thereby sustaining glycolytic metabolism, the “Warburg effect,” and promoting tumor cell survival even under hypoxic stress [12, 14]. Beyond its metabolic role, MCT1 has been implicated in modulating the tumor microenvironment by influencing extracellular acidification and immune cell function [14]. In hepatocellular carcinoma (HCC), MCT1 has drawn particular attention due to its involvement in acetate metabolism and tumor progression [15]. Studies have demonstrated that MCT1 enhances [11C]acetate uptake in HCC cells and that its inhibition markedly suppresses acetate utilization and cell proliferation [15, 16]. Furthermore, high MCT1 expression in liver cancer correlates with increased tumor aggressiveness, metastatic potential, and poor patient prognosis, underscoring its clinical significance [15]. Given these findings, MCT1 is not only viewed as a central player in liver cancer metabolism but also as a promising therapeutic target. Pharmacological inhibition of MCT1, such as with the selective inhibitor AZD3965, is under active investigation and shows potential for disrupting tumor metabolism and improving therapeutic outcomes [16, 17]. Together, these insights suggest that MCT1 as a critical mediator of metabolic reprogramming and disease progression in liver cancer. However, the role of MCT1 in HCC development has not been well established using in vivo models.

To investigate the role of MCT1 in HCC, we generated liver‐specific MCT1 knockout mice. We found that deletion of MCT1 in hepatocytes did not affect liver morphology, proliferation, or apoptosis. Using the DEN/CCl₄ model to induce HCC, we observed that MCT1 knockout did not reduce tumor size, tumor number, proliferation, fibrosis, or inflammation. These findings suggest that MCT1 is dispensable for HCC development and its solo‐deletion is insufficient to mitigate tumor progression in this model.

## 2 Materials and Methods

### 2.1 Mice

All animals received humane care according to the “Guide for the Care and Use of Laboratory Animals” (http://oacu.od.nih.gov/ac_cbt/guide3.htm). The procedures for all animal experiments were approved by the Institutional Animal Care and Use Committee at Loyola University Chicago. All mice were housed in micro‐isolator cages in a room illuminated from 7:00 a.m. to 7:00 p.m. (12:12‐h. light‐dark cycle) and were given access to water and chow ad libitum. MCT1 f/f mice were generated by Applied Stem cells, using CRSPR Cas9 gRNA loxP to flank SLC16A1 gene (Figure 2A). The mice were genotyped using the primers listed in Table 1.

**Table 1:** Genotyping primers.

SLC16A1 F1	5′‐GGGATAAGGTGGTGTAAACTTGG‐3′
SLC16A1 R1	5′‐TTCCAGCTAGACTACAAACCAAGG‐3′
SLC16A1 F2	5′‐GATCTCTCCCTGTAGCACTTGTC‐3′
SLC16A1 R2	5′‐TACTCCAATCACCAAGGCCAAAG‐3′
Cre‐F	5′‐TACCTGGAAAATGCTTCTGT‐3′
Cre‐R	5′‐TGATCTCCGGTATTGAAACT‐3′

### 2.2 DEN/CCl_4_‐Induced HCC Model

Liver‐specific knockouts of MCT1 (AlbCre; MCT1^flox/flox^) mice were generated by crossing AlbCre mice with MCT1 flox/flox (MCT1 f/f) mice. HCC was induced in MCT1 f/f or AlbCre; MCT1 f/f mice, using diethylnitrosamine/Carbon tetrachloride (DEN/CCl_4_) model (Figure 2A), in which 2‐weeks old mice received a single injection of a carcinogen (DEN, ip, 25 mg/kg). After 4‐weeks of DEN injection, mice were ip injected once a week with CCl_4_ (Sigma Aldrich) (1:2 CCl_4_:corn oil), for 15‐consecutive weeks. Then, mice were monitored for the following 10‐weeks to allow HCC tumors growth. In the 11th week, mice were euthanized, and livers were collected, sampled and fixed for histological and immunohistochemical analyses. Additional liver samples were snap frozen on dry ice and stored at −80°C for further examination.

### 2.3 Western Blot Analysis

Western blot analysis was performed as previously described [18, 19]. Information on primary antibodies is shown in Table 2. Briefly, Liver tissues were digested by RIPA buffer (Sigma) containing phosphatase and protease inhibitors (Thermo‐Fisher). Total protein lysates (10 μg) were loaded onto a 10% SDS‐PAGE gel and subsequently transferred to a nitrocellulose membrane. Following blocking with 5% BSA, membranes were probed with antibodies against MCT1 (Santa Cruz), and GAPDH (Cell signaling) was used as a housekeeping gene. All images were acquired using iBright imaging system (Thermo‐Fischer Scientific).

**Table 2:** Antibodies.

Protein	Species	Catalog number	Source	Application
Alpha‐1‐fetoprotein (AFP)	Rabbit	a0008	Dako	IHC
Alpha smooth muscle actine (ɑ‐SMA)	Rabbit	I9245s	Cell Signaling	IHC
Collagen A1 (COLA1)	Rabbit	72026	Cell Signaling	IHC
GAPDH	Mouse	MA5‐15738	Invitrogen	Western Blot
Ki‐67	Rabbit	12202	Cell Signaling	IHC
Monocarboxylate Transporter‐1 (MCT1)	Mouse	sc‐365501	Santa Cruz	Western Blot
Myeloperoxidase (MPO)	Rabbit	ab208670	Abcam	IHC

### 2.4 Quantitative Real‐Time Polymerase Chain Reaction (RT‐qPCR)

Total RNA was extracted from liver tissue using Zymo Quick‐RNA kits (Irvine, CA) and quantified by Nanodrop 1000 (Thermo‐Fischer Scientific). cDNA was reverse‐transcribed using Bio‐Rad iScript cDNA synthesis kit. Quantitative PCR was performed using SYBR green on QuantStudio 6 Real‐Time PCR System (Thermo‐Fisher, Waltham, MA). The sequences of primers are listed in Table 3.

**Table 3:** qPCR primers.

SLC16A1‐F	5′‐TGTTAGTCGGAGCCTTCATTTC‐3′
SLC16A1‐R	5′‐CACTGGTCGTTGCACTGAATA‐3′
GAPDH‐F	5′‐CTCTGGAAAGCTGTGGCGTGATG‐3′
GAPDH‐R	5′‐ATGCCAGTGAGCTTCCCGTTCAG‐3′

### 2.5 Hematoxylin & Eosin (H&E) Staining

Liver tissues were fixed in zinc‐buffered formalin overnight, then tissue cassettes were stored in 70% ethanol for embedding. The formalin‐fixed paraffin‐embedded (FFPE) tissues were sectioned at 5 µm and stained with hematoxylin and eosin. The H&E‐stained images were acquired using microscope.

### 2.6 Immunohistochemical (IHC) Staining

IHC staining of FFPE mouse liver tissue was performed as previously described [20]. Paraffin‐embedded liver tissue Section (5 µm) were subjected to deparaffinization, hydration and antigen retrieval. Then, primary antibodies (Table 2) were used against alpha‐fetoprotein (AFP), Ki67, myeloperoxidase (MPO), alpha smooth muscle actin (αSMA), and collagen A1 to detect their protein expression in liver tissues. Antibody binding was visualized as brown color by reaction of the coupled peroxidase with 3,3′‐diaminobenzidine (DAB Substrate Kit Peroxidase; Vector Labs, SK‐4100), as recommended by the manufacturer. Tissue sections were counterstained with hematoxylin and mounted. Images were acquired using microscope and protein expression was quantified using FIJI Image J software.

### 2.7 Statistical Analysis

All statistical significance analyses in this study were used by GraphPad Prism software. Data are expressed as means ± SEM from at least three independent experiments except where otherwise indicated. Differences between groups were determined by a two‐tailed Student's *t*‐test. *p* < 0.05 was considered significant. TCGA LIHC cohort data were analyzed using GEPIA2 [21] or the Human Protein Atlas.

## 3 Results

### 3.1 High Expression of MCT1 Is Correlated With Poor Prognosis in Human HCC Samples

**Figure 1:**
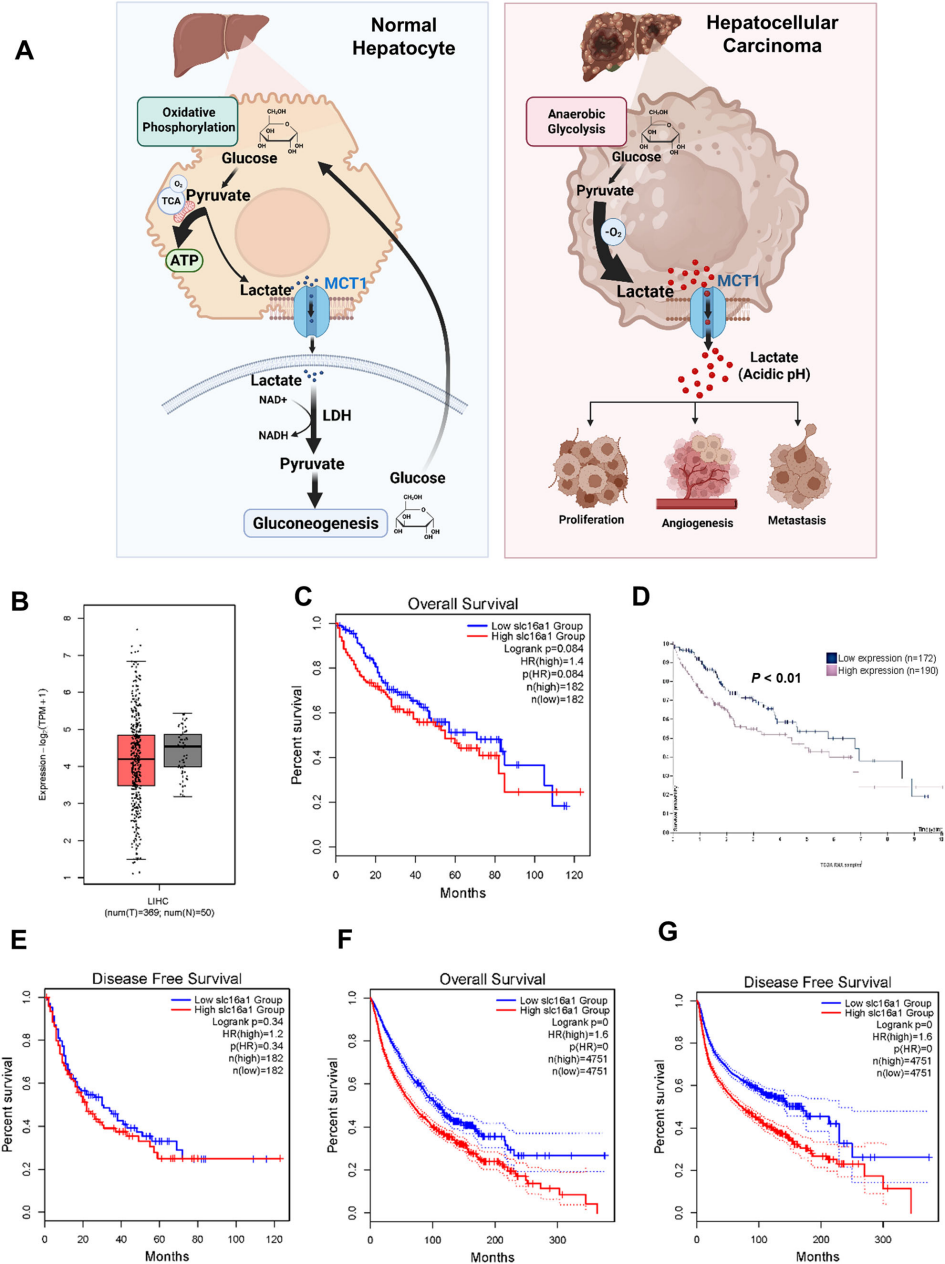
High expression of MCT1 in human HCCs is positively correlated with shorter survival times of patients. (A) A schematic diagram of MCT1's function in cancer development. Created with BioRender. (B) Relative expression of MCT1 mRNA in normal liver and HCC specimens from the TCGA LIHC database was analyzed using GEPIA2. (C) Correlation of MCT1 mRNAs with patient overall survival in HCC samples from the TCGA LIHC database was analyzed using GEPIA2. (D) Correlation of MCT1 mRNAs with patient overall survival in HCC samples from the TCGA LIHC database was analyzed using the human protein atlas. (E) Correlation of MCT1 mRNAs with patient disease‐free survival in HCC samples from the TCGA LIHC database was analyzed using GEPIA2. (F) Correlation of MCT1 mRNAs with patient overall survival in all cancer type samples from the TCGA database was analyzed using GEPIA2. (G) Correlation of MCT1 mRNAs with patient disease‐free survival in all cancer type samples from the TCGA database was analyzed using GEPIA2.

An earlier study showed that MCT1 expression was increased in tumor tissues than adjacent normal tissues [22]. Using TCGA LIHC sample cohort, we observed that MCT1 mRNA levels are not increased in tumor samples compared to normal liver controls (Figure 1B). However, when HCC tumor samples were stratified by MCT1 mRNA levels, patients with high MCT1 expression had worse overall survival (Figure 1C,D), although high expression of MCT1 was not significantly correlated with disease‐free survival (Figure 1E). Notably, high expression of MCT1 was correlated with poor prognosis across all cancer types in the TCGA database (Figure 1F,G).

### 3.2 Normal Liver Morphology, Histology or Cellular Proliferation Was Maintained in Liver Specific MCT1‐Deleted Mice

**Figure 2:**
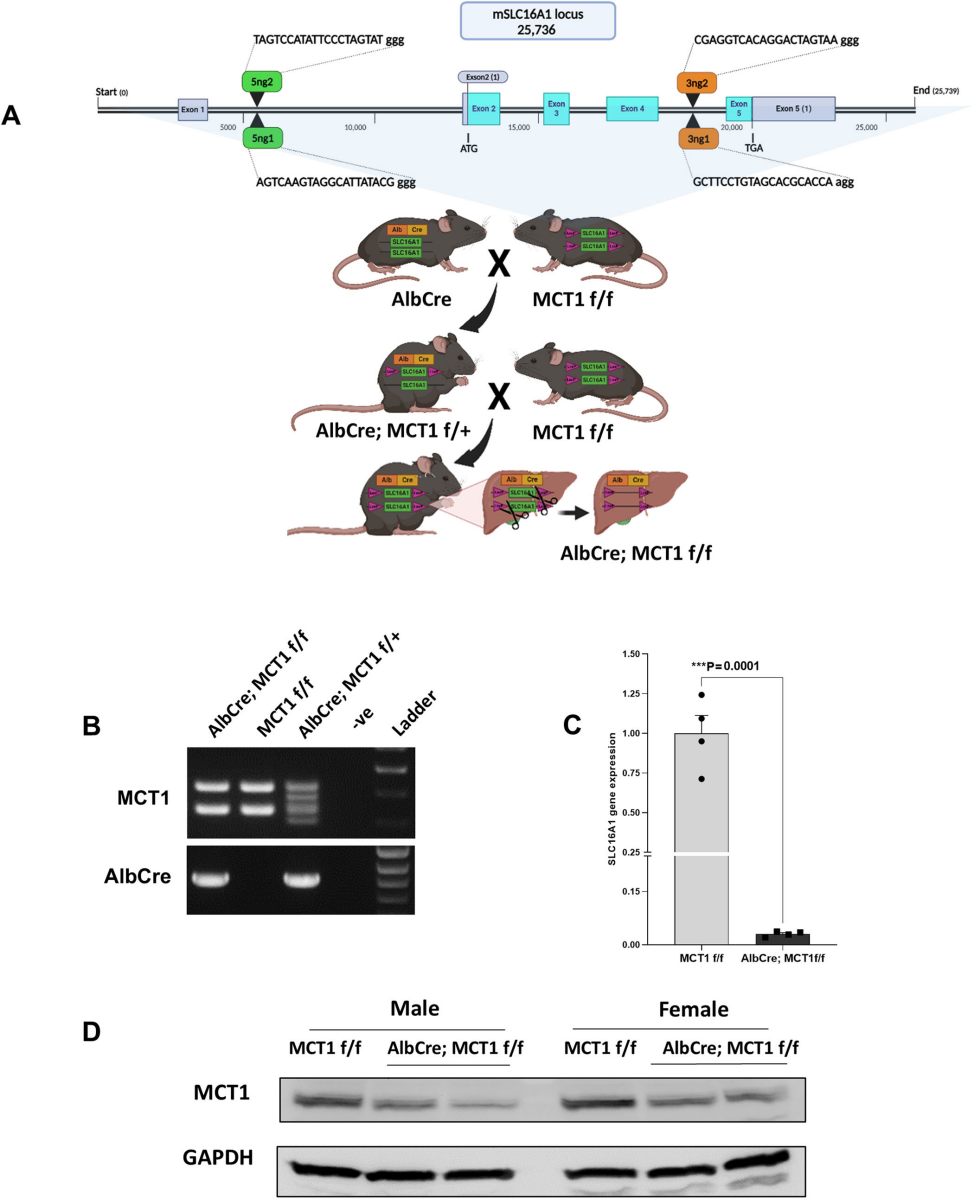
Characterization of MCT1 deletion in liver. (A) A schematic diagram of the generation of MCT1 f/f and AlbCre; MCT1 f/f mice. (B) Genotyping analysis to confirm the Alb‐Cre driven MCT1 knockout mouse model by PCR. (C) qPCR analysis confirming the deletion of SLC16A1 (MCT1 gene) in liver cells. Significance was assessed using Student′s t‐test. ***p < 0.001. (D) Western blot analysis confirming the deletion of MCT1 in mouse livers.

**Figure 3:**
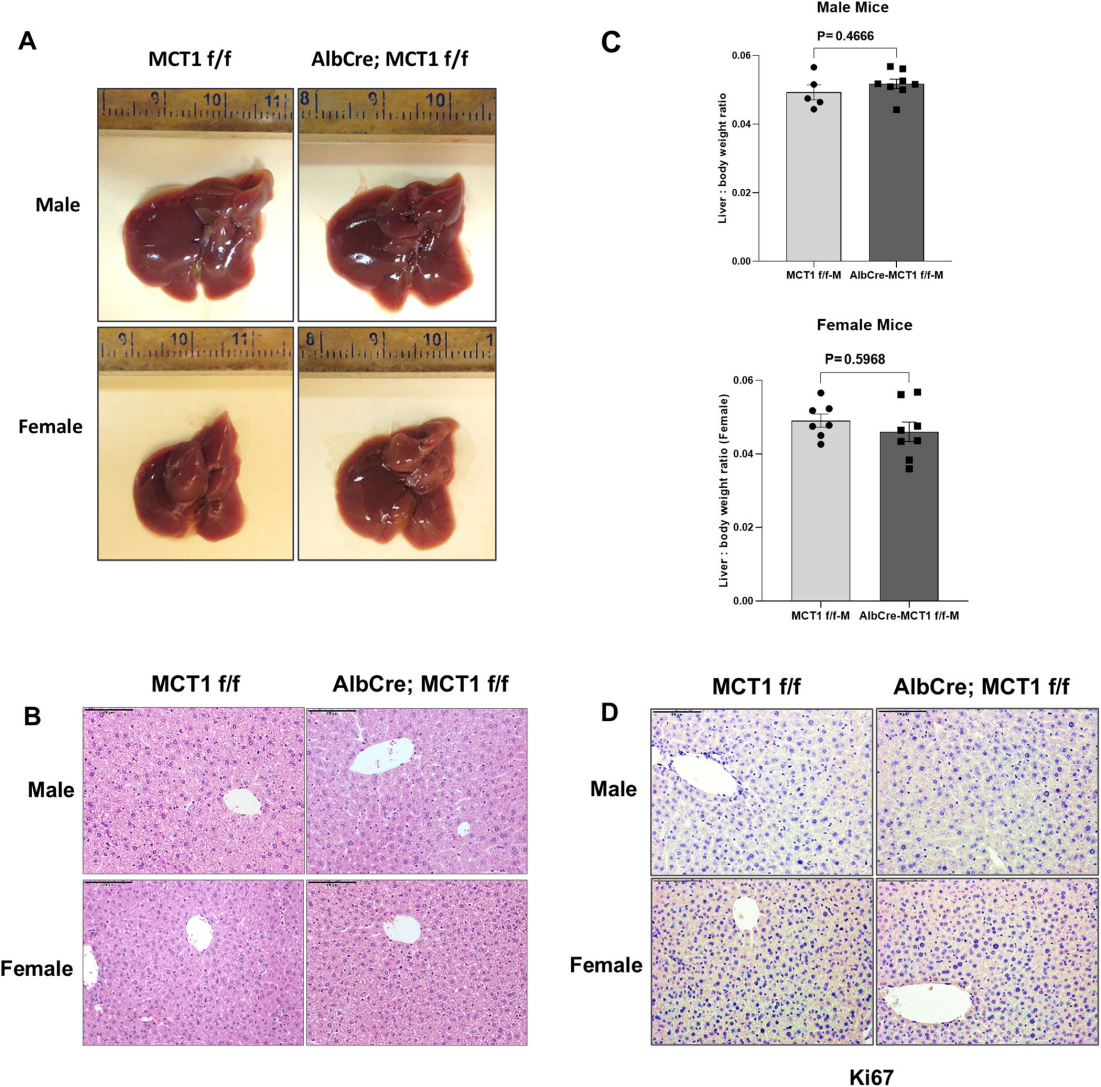
Deletion of MCT1 in hepatocytes does not affect morphology, histology or proliferation in mouse liver. (A) Representative liver images from nontreated MCT1 f/f and AlbCre; MCT1 f/f mice. (B) H&E staining of liver tissue from nontreated MCT1 f/f and AlbCre; MCT1 f/f mice. (C) Liver‐to‐body weight ratio from nontreated MCT1 f/f and AlbCre; MCT1 f/f mice. (D) Immunohistochemical analysis of Ki‐67 expression in liver tissue from nontreated MCT1 f/f and AlbCre; MCT1 f/f, assessing cell proliferation.

To investigate the role of MCT1 in liver tumorigenesis, we first generated MCT1^flox/flox^ (MCT1 f/f) mice using Cas9/CRISPR technology (Figure 2A). We then generated liver‐specific MCT1 knockout mice by crossing AlbCre mice and MCT1 f/f mice. AlbCre; MCT1 f/f mice were compared to MCT1 f/f only control mice. We confirmed that the expression of Cre recombinase in liver removes the MCT1 allele between two loxP sites by PCR (Figure 2B). We also confirmed the knockout of MCT1 in the liver by qPCR and western blot, with a significant reduction in MCT1 at both the mRNA and protein levels (Figure 2C,D). AlbCre; MCT1 f/f mice are viable, fertile, and indistinguishable from MCT1 f/f mice, suggesting that MCT1 is not required for normal liver development. There was no significant difference in morphology and histology of livers between AlbCre; MCT1 f/f and MCT1 f/f mice by gross evaluation (Figure 3A) or H&E staining (Figure 3B), and there was no significant difference in liver‐to‐body weight ratio between AlbCre; MCT1 f/f and MCT1 f/f mice (Figure 3C). Furthermore, the knockout of MCT1 did not affect cell proliferation in mouse liver, by Ki‐67 IHC (Figure 3D). These results suggest that deletion of MCT1 does not affect mouse liver morphology, histology, and proliferation.

### 3.3 Liver‐Specific Deletion of MCT1 Does Not Affect DEN/CCl_4_‐Induced Hepatocarcinogenesis

**Figure 4:**
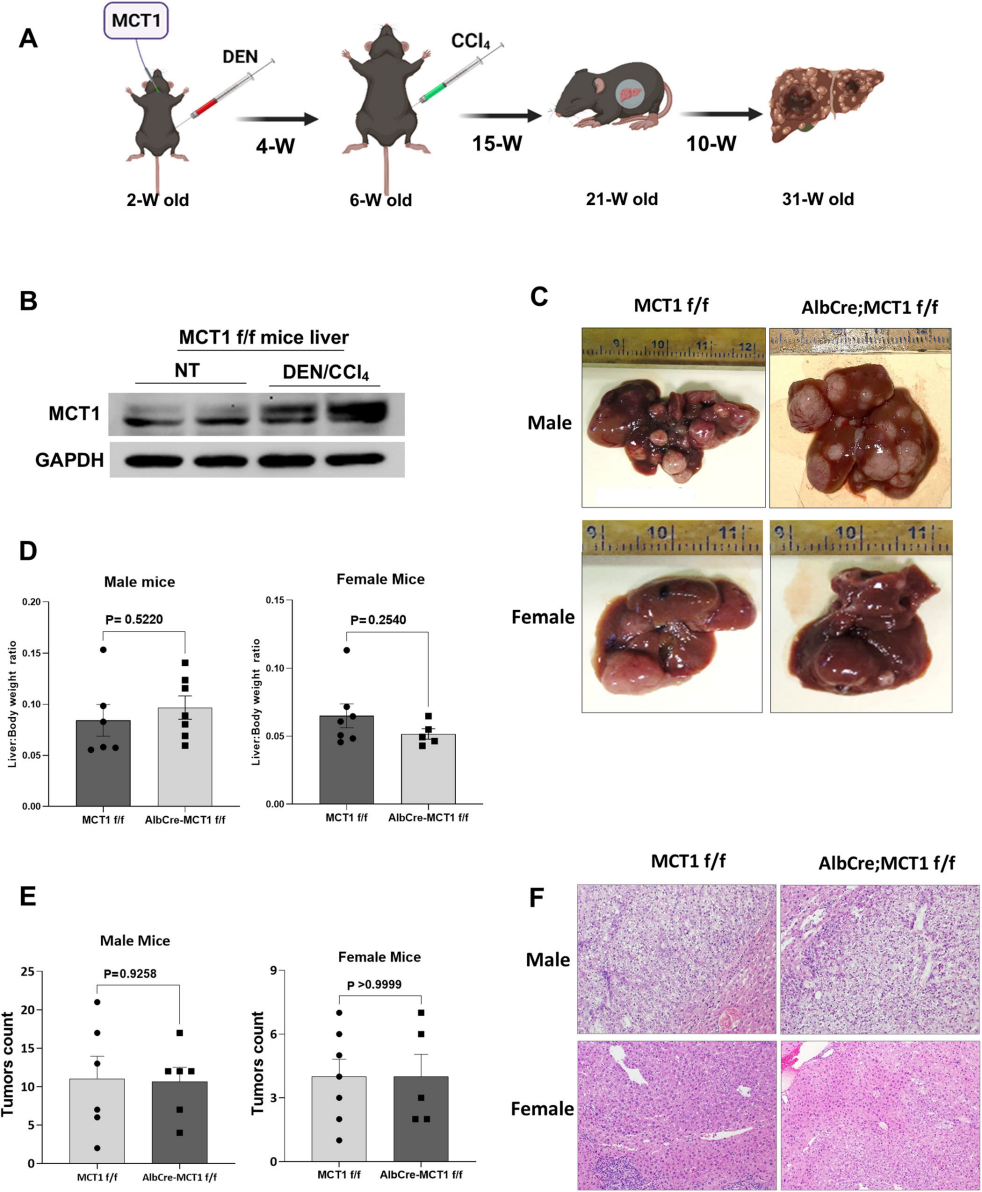
Deletion of MCT1 does not affect morphology, histology, or tumor count in DEN/CCl_4_‐treated male mice. (A) A schematic diagram of DEN/CCl_4_ model for the induction of HCC. (B) Western blot analysis of MCT1 expression in normal liver tissue and DEN/CCl_4_‐induced liver tumors. (C) Representative liver images extracted from male and female MCT1 f/f and AlbCre; MCT1 f/f mice at 31 weeks posttreatment of DEN/CCl_4_. (D) Tumor burden presented by the liver‐to‐body weight ratio in DEN/CCl_4_‐treated MCT1 f/f and AlbCre; MCT1 f/f mice. Significance was assessed using Student's *t*‐test. (E) Tumor burden presented by the tumor counts per mouse in MCT1 f/f and AlbCre; MCT1 f/f mice. Significance was assessed using Student's *t*‐test. Each bar represents mean ± SEM of tumors count (*n* = 5–7 mice). (F) H&E staining of liver sections from male and female MCT1 f/f and AlbCre; MCT1 f/f mice at 31 weeks posttreatment of DEN and CCl_4_.

To investigate the effect of MCT1 deletion on the development of HCC in immune‐competent mice, we used a well‐established DEN/CCl_4_‐induced HCC model [23] in both male and female mice (Figure 4A). First, we found that MCT1 expression was increased in DEN/CCl_4_‐driven HCC tumors compared to normal nontreated (NT) liver controls (Figure 4B). Further, we found that comparable tumor growth in AlbCre; MCT1 f/f and MCT1 f/f both male and female mice (Figure 4C–E). It was notable that more and bigger tumors were observed in male mice than female mice (Figure 4D–F).

### 3.4 Liver‐Specific Deletion of MCT1 Was Not Sufficient to Alleviate the Pathological Repercussions Associated With DEN/CCl_4_‐Induced HCC

**Figure 5:**
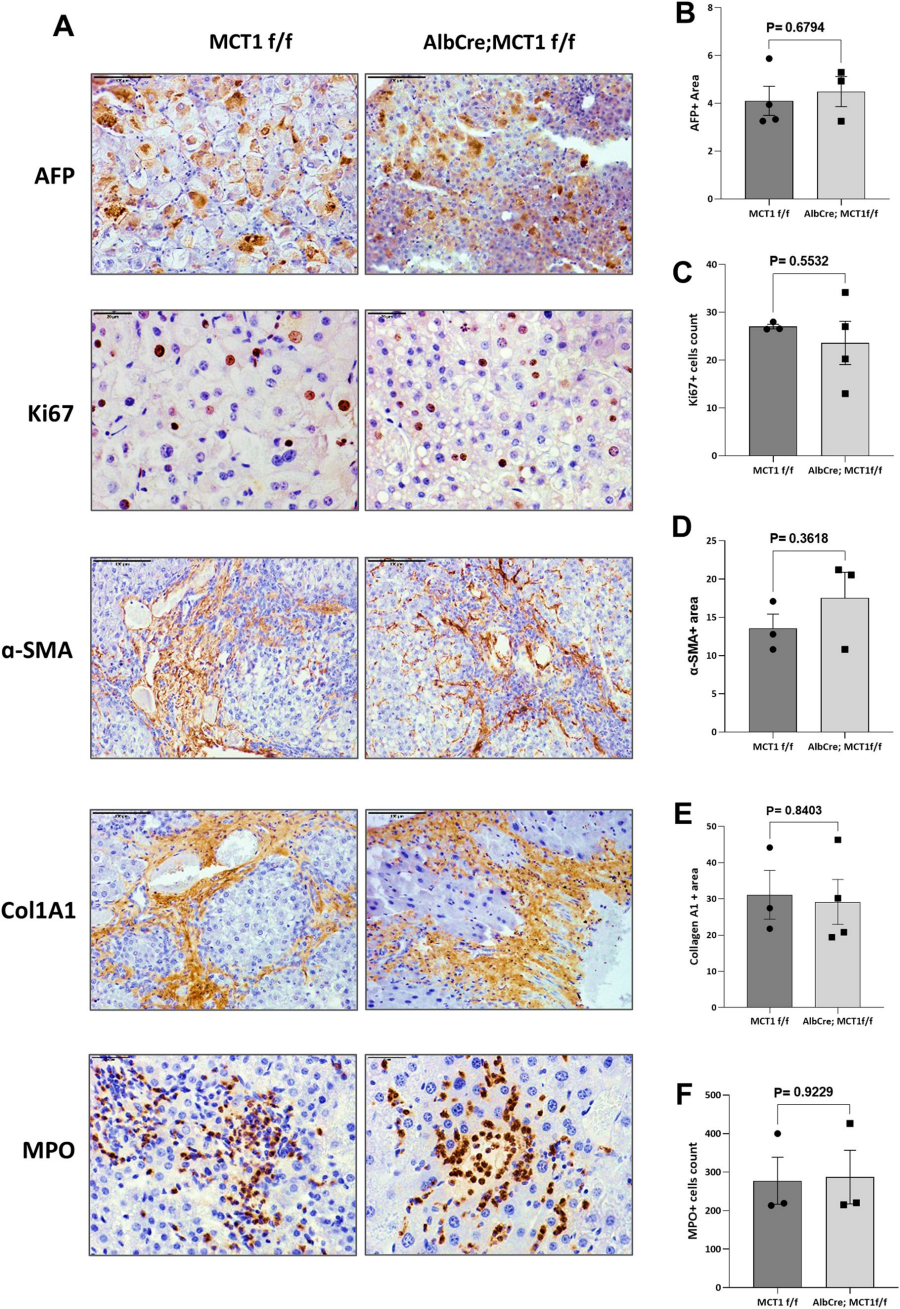
Liver‐specific deletion of MCT1 was not sufficient to alleviate the pathological repercussions associated with DEN/CCl_4_‐induced HCC. (A) AFP, Ki‐67, α‐SMA, COLA1 and MPO immunohistochemical staining analysis of the liver sections of MCT1 f/f and AlbCre; MCT1 f/f mice at 31 weeks posttreatment of DEN/CCl_4_ (20× magnification). (B) Quantification of AFP staining from 5A. AFP positive staining was scored in at least three fields (20× magnification) per mouse, reported as mean ± SEM. Significance was assessed using Student's *t*‐test. ns: *p* > 0.05. (C) Quantification of Ki‐67 staining from 5A. Ki‐67 positive staining was scored in at least three fields (20× magnification) per mouse, reported as mean ± SEM. Significance was assessed using Student's *t*‐test. ns: *p* > 0.05. (D) Quantification of α‐SMA staining from 5A. α‐SMA positive staining was scored in at least three fields (20× magnification) per mouse, reported as mean ± SEM. Significance was assessed using Student's *t*‐test. ns: *p* > 0.05. (E) Quantification of ColA1 staining from 5A. ColA1 positive staining was scored in at least three fields (20× magnification) per mouse, reported as mean ± SEM. Significance was assessed using Student's *t*‐test. ns: *p* > 0.05. (F) Quantification of MPO staining from 5A. MPO positive staining was scored in at least three fields (20× magnification) per mouse, reported as mean ± SEM. Significance was assessed using Student's *t*‐test. ns: *p* > 0.05.

**Figure 6:**
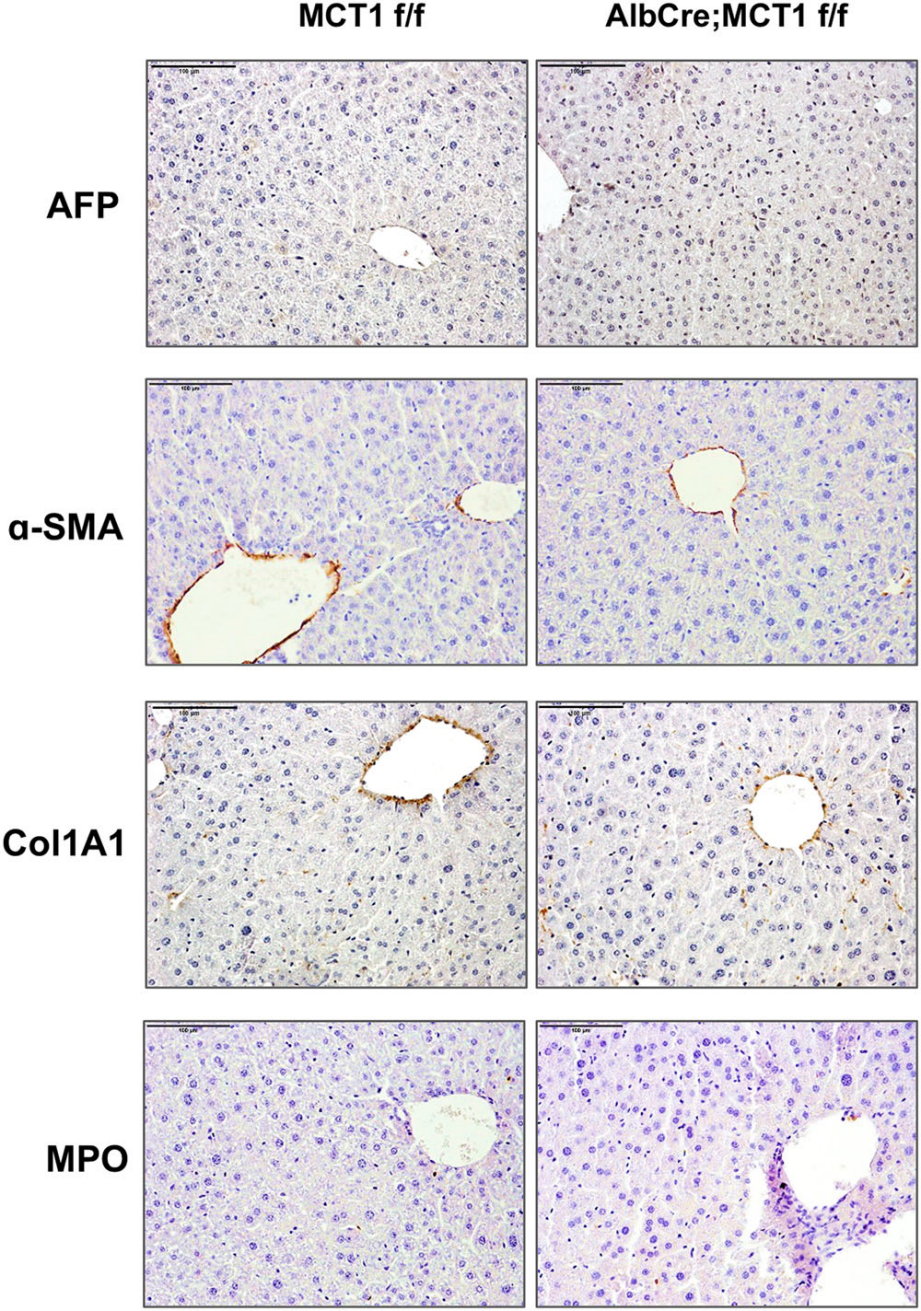
Liver‐specific deletion of MCT1 does not induce proliferation, fibrosis or inflammation in mouse liver. IHC staining analysis of AFP, Ki‐67, α‐SMA, COLA1 and MPO using paraffin‐embedded liver sections from nontreated MCT1 f/f and AlbCre; MCT1 f/f mice (20× magnification).

To evaluate the pathological features associated with DEN/CCl_4_‐induced HCC, IHC staining was utilized to detect the expression of HCC‐related proteins. The staining results showed that DEN/CCl_4_ treatment induced HCC tumors associated with fibrosis and inflammation, where high expression levels of related markers were detected in DEN/CCl_4‐_treated (Figure 5A), compared to low or undetectable levels in nontreated mice (Figure 6). Alpha fetoprotein (AFP) was used as HCC tumor marker, and the staining results revealed that comparable levels of AFP in DEN/CCl_4_‐treated AlbCre; MCT1 f/f and MCT1 f/f mice (Figure 5A,B), indicating that liver‐specific MCT1 deletion was not sufficient to modify the severity of DEN/CCl_4_‐induced HCC tumors. Ki67 staining, a proliferation marker, was also not changed by MCT1 deletion (Figure 5A,C), indicating that hyper‐proliferation was not tackled by MCT1 deletion in DEN/CCl_4_‐induced hepatocarcinogenesis. In addition, HCC‐associated fibrosis was evaluated by collagen A1 and α‐SMA expression, which was not affected by MCT1 deletion (Figure 5A,D,E). In addition, Myeloperoxidase (MPO), a marker and mediator of inflammation, showed a similar expression pattern in AlbCre; MCT1 f/f and MCT1 f/f ^flox^ mice (Figure 5A,F). Taken together, liver‐specific deletion of MCT1 is insufficient to affect the pathological repercussions associated with DEN/CCl_4_‐induced HCC.

## 4 Discussion

In this study, we investigated the functional role of MCT1 in HCC by employing a liver‐specific knockout model. Despite MCT1 has a well‐established role in lactate and acetate transport, and its proposed importance in tumor metabolism [10, 11], our findings demonstrate that liver‐specific deletion of MCT1 does not impact liver morphology, cell proliferation, or apoptosis under physiological conditions. More importantly, in the context of chemically induced liver carcinogenesis using the DEN/CCl₄ model, MCT1 deletion did not alter tumor burden, histopathological features, or key indicators of tumor‐associated proliferation, fibrosis, or inflammation.

This finding is somewhat unexpected given the growing literature implicating MCT1 in cancer progression. Several studies have reported that MCT1 expression is elevated in human HCC and that increased acetate uptake via MCT1 correlates with poor prognosis [22]. Functional studies in vitro have also demonstrated that MCT1 inhibition can impair tumor cell proliferation and survival under nutrient‐depleted conditions [16, 22]. However, our in vivo data suggest that these in vitro dependencies may not fully translate to the complex tumor microenvironment of the liver, or that MCT1 function may be compensated for by other mechanisms in vivo.

One potential explanation for this discrepancy is metabolic redundancy. The liver expresses multiple monocarboxylate transporters, including MCT2 and MCT4, which may partially or fully compensate for the loss of MCT1 [24]. Alternatively, non‐hepatocyte populations within the tumor microenvironment—such as endothelial cells, stellate cells, or immune cells—may play more dominant roles in metabolite exchange and buffering, thereby diminishing the impact of hepatocyte MCT1 deletion. Furthermore, the metabolic demands of HCC may be fulfilled through enhanced glucose or amino acid metabolism, reducing reliance on monocarboxylate substrates under the conditions used in our study.

Another important consideration is context dependency. While the DEN/CCl₄ model mimics chronic liver injury and fibrosis‐driven tumorigenesis, it may not fully capture the diversity of metabolic states observed in human HCC. For example, metabolic reliance on MCT1 may be more pronounced in tumors driven by distinct oncogenes, in specific molecular subtypes, or under conditions of metabolic stress such as fasting, hypoxia, or high‐fat diet exposure. Indeed, prior studies using PET tracers have shown variable acetate uptake in HCCs [25], suggesting metabolic heterogeneity that may influence the degree to which MCT1 contributes to tumor growth.

From a therapeutic standpoint, our findings suggest that MCT1 inhibition alone may not be sufficient to prevent or reverse HCC progression, at least when targeting hepatocytes. This has important implications for drug development strategies that aim to disrupt tumor metabolism by targeting lactate or acetate transport. It also highlights the need for biomarker‐driven approaches to identify patient subsets who may derive benefit from such interventions. Given the complexity and plasticity of cancer metabolism, combination strategies targeting multiple metabolic pathways or tumor cell types may be required to achieve meaningful therapeutic effects.

In conclusion, our study provides in vivo evidence that MCT1 is not required for liver tumorigenesis in the DEN/CCl₄ model of HCC. While MCT1 remains an attractive metabolic target in certain cancers, its role in HCC appears to be context‐dependent and potentially compensable. Future studies should explore the role of MCT1 under alternative metabolic conditions, in additional tumor models, and across different cellular compartments within the liver tumor microenvironment. Understanding these nuances will be key to translating metabolic vulnerabilities into effective therapeutic interventions for HCC.

## Author Contributions

Shaimaa A. Gad designed and performed experiments, analyzed data, and wrote the paper. Bryan Bridgeman bred the mice and performed experiments. Kyle Boedeker bred the mice. Xianzhong Ding analyzed data. Wei Qiu designed experiments, analyzed data, and wrote the paper.

## Conflicts of Interest

The authors declare no conflicts of interest.

## Data Availability

The data that support the findings of this study are available from the corresponding author upon reasonable request.
